# Absenteeism during Menstruation among Nursing Students in Spain

**DOI:** 10.3390/ijerph17010053

**Published:** 2019-12-19

**Authors:** Elia Fernández-Martínez, María Dolores Onieva-Zafra, Ana Abreu-Sánchez, Juan José Fernández-Muñóz, María Laura Parra-Fernández

**Affiliations:** 1 Department of Nursing, University of Huelva, 21004 Huelva, Spain; elia.fernandez@denf.uhu.es (E.F.-M.); abreu@denf.uhu.es (A.A.-S.); 2 Department of Nursing, Physiotherapy and Occupational Therapy, University of Castilla-La-Mancha, Ciudad Real, 13071 Ciudad Real, Spain; marialaura.parra@uclm.es; 3 Department of Psychology, Universidad Rey Juan Carlos, 28922 Alcorcón, Madrid, Spain; juanjose.fernandez@urjc.es

**Keywords:** absenteeism, dysmenorrhea, menstrual experiences

## Abstract

Absenteeism can clearly have a negative impact on academic performance among university students. Certain experiences or symptoms such as menstrual pain are very common in women and can lead to absenteeism. The current study was aimed at examining the presence of menstrual experiences or symptoms and their impact upon absenteeism among healthy (illness-free) female university nursing students in Spain. A total of 299 students participated in this research, which was a descriptive cross-sectional, observational study. An ad hoc online questionnaire was used based on sociodemographic and gynecological data, together with the noted menstrual experiences; the most prevalent of which were bloating, which affected 87.3% of students; dysmenorrhea and irritability, which affected 76.3%; and fatigue, which affected 70.6%. Students with dysmenorrhea had a 6.95 higher (odds ratio (OR) 6.95; 95% confidence interval (CI) 3.39–14.25) odds of absenteeism; in those who reported dizziness, the odds of absenteeism was 4.82 times higher (OR 4.82; 1.76–13.23); in those who manifested nausea and vomiting, the percentage of absenteeism was 3.51 higher (OR 3.51; 95% CI 1.51–8.15); in those who presented sleep alterations, the odds were 2.95 higher (OR 2.95; 95% CI 1.39–6.25); and for those who felt depressed the odds were 2.18 times higher (OR 2.18; 95% CI 1.21–3.94) Absenteeism was found to be more likely in women with dysmenorrhea. However, in addition, higher odds of absenteeism were also found in women with nausea and vomiting, dizziness, sleep disorders, and those who feel depressed. These menstrual experiences can be considered a relevant problem among young women, leading to absenteeism, and a negative influence on academic performance. It is essential to raise awareness of the socioeconomic impact of absenteeism and establish new strategies for improving menstrual experiences.

## 1. Introduction 

The primary menstrual symptom or negative experience identified is menstrual pain that is not associated with pathology, known as primary dysmenorrhea, which is estimated to affect between 60%–90% of female university students worldwide [1,2]. In Spain, a recent study estimated that this affects approximately 75% [3] of this population. Many studies have identified how pain can affect people, for example, headaches are known to decrease the academic performance of university students [4]. More concretely, menstrual pain increases absenteeism and decreases academic performance [5,6]. However, most previous studies are based on studying dysmenorrhea alone, while few studies have focused on analyzing other menstrual experiences in depth, such as fatigue, nausea, diarrhea, headache, dizziness, feeling depressed, sleep disorders, or irritability. Even in these cases, studies have tended to concentrate on the teenage age group [7,8,9,10].

Absenteeism is a problem which can negatively affect the academic performance of students [11,12,13]. Previous studies have shown that nursing students’ regular attendance in classes or at hospital placements improved their academic performance, whereas absenteeism was related to worse results [14,15]. Therefore, studies examining the reasons for absenteeism among students are clearly relevant.

The university training of health professionals and, more concretely, that of nursing professionals requires an extensive dedication to mandatory clinical practice. In Spain, according to the European Directives of the European Higher Education Area (EHEA), a minimum of 2300 hours of clinical practice is a mandatory requirement [16]. Therefore, this requires monitoring the attendance of health professionals at their respective higher education center. Nonetheless, due to the estimation of what is considered moderate absenteeism, the programs themselves have established procedures regarding how to make up for clinical placements when students are absent. The nursing studentship is primarily female and of a childbearing age. In addition, primary dysmenorrhea and some other experiences or symptoms related with menstruation have already been shown to affect a large part of this collective of adolescents and young women [17,18].

The aim of the present study was to examine experiences or symptoms related with menstruation and their impact on absenteeism among healthy (illness-free) university nursing students in Spain.

## 2. Materials and Methods

### 2.1. Design, Setting, and Participants

**Figure 1:**
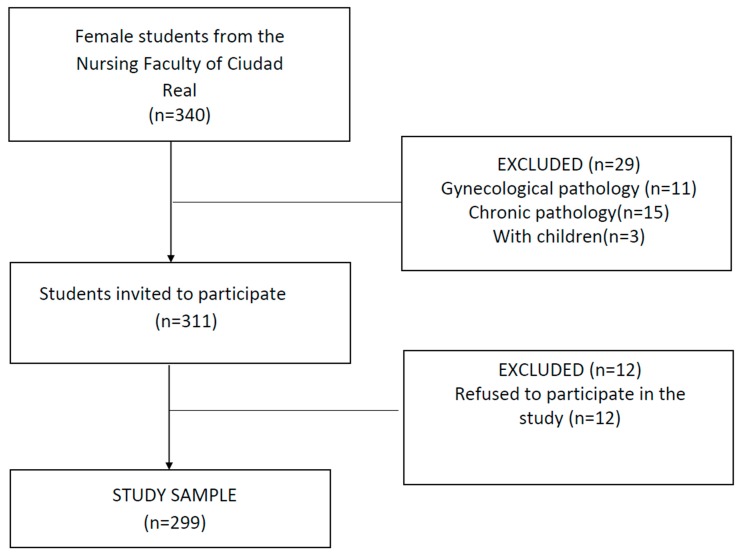
Flowchart of study participants.

A cross sectional observational descriptive study was performed among university students at the University of Castilla-La Mancha (Ciudad Real Campus, Spain). The exclusion criteria were being under the age of 18, suffering from a diagnosed gynecological illness (endometriosis, polycystic ovary syndrome), suffering some kind of chronic illness, or having undergone childbirth. All students over the age of 18 years who enrolled in the academic year 2017/2018 at the above-noted nursing faculty were invited to participate in this study. A flow chart of study participants is shown in Figure 1, according to STrengthening the Reporting of OBservational studies in Epidemiology (STROBE) guidelines [19,20]. A teacher who was not involved in the research invited students to participate voluntarily in the classrooms for data collection. The invitation to participate took place on three different days on different weeks during the month of May 2018. When the classes concluded, the students interested in participating were accompanied by their teacher to the computer lab to complete an on-line questionnaire using the computers available at the faculty. The data were gathered via the internet using the JotForm platform. An ad hoc questionnaire was used for this study, which included sociodemographic characteristics, gynecological characteristics, and menstrual symptoms (Supplementary file 1). A Visual Analogic Scale (VAS scale) was included to score the intensity of menstrual pain from 0 to 10, as employed in former studies on dysmenorrhea [3,21,22]. Prior to gathering the data, cognitive interviews were conducted with five university students to test comprehensibility and readability of the questionnaire.

Considering that the central study theme is absenteeism, the considerations by Young et al. were considered during the study regarding the ethical and methodological challenges of studying absenteeism by surveying students who attend university; therefore, the surveys were performed on different days in different weeks [23].

The study was performed by respecting the principles of the Helsinki Declaration at all times, obtaining the informed consent of participants and treating data in an anonymized manner. The study protocol was approved by the Clinical Research Ethics Committee of the General Hospital of Ciudad Real (approval number C-105).

### 2.2. Data Analysis

The data were entered into an Excel Microsoft Office package and analyzed using SPSS for Windows version 23.0 (Statistical Package for Social Sciences Inc., Chicago, IL, USA). The data were examined by calculating the mean, the standard deviation from quantitative variables, and the frequency and the percentage for qualitative data. The Pearson’s chi-squared test and the Student’s t-test were used for the bivariate analyses, comparing the women who reported menstrual absenteeism and those who did not, in relation to the gynecological variables and menstrual experiences or symptoms they suffered. For variables where the normal distribution was *p* < 0.05, the Mann–Whitney U Test was applied, as a nonparametric test. Lastly, a backward stepwise binary logistic regression was performed to predict absenteeism on the basis of menstrual experiences. The significance level was set at *p* < 0.05, with a 95% confidence interval.

## 3. Results

The response rate was 82.6%. In total, 299 university women fulfilled the inclusion criteria, with a mean age of 20.12 ± 2.40 years. The mean weight of respondents was 58.69 ± 10.2 kg, the height was 164.46 ± 6.32 cm, and the body mass index (BMI) was 21.67 ± 3.24 (Table 1).

### 3.1. Absenteeism and Sociodemographic and Menstrual Characteristics

A total of 60.5% (181) declared having had to miss classes during menstruation due to different aspects related to the over the previous year. However, no differences were found regarding absenteeism by age (*p* = 0.80) nor by BMI (*p* = 0.51).

Most women presented normal menstrual characteristics: duration of flow lasting 3–7 days (97.3%) and a cycle lasting 21 to 35 days (88.3%); however, 66.9% reported having irregular cycles (see Table 2).

Among the women who used hormonal combined oral contraceptives (11.71%), there were less cases of absenteeism related with menstruation, compared with those who did not use the same (*p* = 0.021). However, no differences were found when analyzing the remaining menstrual characteristics, together with absenteeism during menstruation. The mean number of days of flow per menstruation was 4.97 ± 1.26 in women who did not report absenteeism, compared with 5.22 ± 1.28 in women who did (*p* = 0.110), and the mean duration of the cycle was 30.36 ± 5.00, compared with 30.35 ± 7.02 (*p* = 0.889). Regarding the mean age of menarche in women who reported absenteeism during menstruation days, this was similar to those who did not manifest the same (12.30 ± 1.43 compared to 12.49 ± 1.45, *p* = 0.253).

### 3.2. Absenteeism and Menstrual Experiences or Symptoms

The most prevalent experiences or symptoms during menstruation were bloating, affecting 87.3%, and dysmenorrhea and irritability, which affected 76.3%, and fatigue, which affected 70.6% of all the women under study. The least prevalent experiences were dizziness (22.7%), sleep disorders (25.4%), and nausea and vomiting (25.8%).

The proportion of women referring absenteeism during menstruation was statistically higher to those who suffered dysmenorrhea (Chi squared X^2^ = 69.48, Contingency coefficient C = 0.434, *p* < 0.01) and also for those who reported dizziness, nausea and vomiting, sleep disorders, fatigue, bloating, headache, irritability, and depression. When comparing women with and without dysmenorrhea, a greater proportion of women were found with bloating, irritability, fatigue, dizziness, sleep disorders, and nausea and vomiting among women who did suffer dysmenorrhea (see Table 3).

As the scores of the VAS were normally distributed (*p* < 0.05), the Mann–Whitney U test was used to determine differences. Among the group of women who did suffer dysmenorrhea, higher rates of absenteeism were found among students who manifested a greater intensity of the same according to the VAS scale, with a mean rank (MR) of 172.59, compared with MR = 81.51 (U = 14,795.00, *p* < 0.01).

Up to 91.7% of women manifested suffering several menstrual symptoms simultaneously, the number of mean menstrual symptoms or negative experiences in women who did not suffer absenteeism was lower (3.88 ± 1.91) than in those who did manifest the same (6.27 ± 1.87), (*p* = 0.000).

All the women who suffered dysmenorrhea (i.e., 100% (228)) manifested some other negative experience or symptom during menstruation, the most frequent of which were bloating 91.67% (209); irritability 81.14% (185), fatigue 78.07% (178), and depression 65.79% (150). However, we found that 21.4% (64) of the total sample under study did not suffer dysmenorrhea, although they did suffer some other symptom or negative experience during menstruation. Among the group of women who did not suffer dysmenorrhea (71), they commonly manifested bloating 73.24% (52), irritability 60.56% (43), fatigue 46.48% (33), and depression 42.25% (30). Besides, 18.31% (13) of these women also acknowledged absenteeism at some point over the last year during their menstruation due to one of these experiences associated to menstruation, although they did not present dysmenorrhea. In order of prevalence, the negative symptoms or experiences reported by these students without dysmenorrhea during menstruation and which affected their attendance were bloating 84.6% (11); irritability 69.2% (9); feeling depressed 61.5% (8); fatigue 61.5% (8); diarrhea 38.5% (5); headache 30.8% (4); sleep disorders 15.4% (2); dizziness 7.7% (1); and nausea and vomiting 7.7% (1). Of these, only 15.4% (2) declared that they were taking hormonal contraceptives.

The results of the backward stepwise binary logistic regression using the significant bivariate variables are shown in Table 4. The odds of absenteeism in participants with dysmenorrhea were 6.95 higher (OR 6.95; 95% CI 3.39–14.25), in those who presented dizziness, this was 4.82 (OR 4.82; 95% CI 1.76–13.23), whereas in those who manifested nausea and vomiting, it was 3.51 higher (OR 3.51; 95% CI 1.51–8.15), among those who presented sleep disorders, it was 2.95 higher (OR 2.95; 95% CI 1.39–6.25), and for those who felt depressed, the odds were 2.18 times higher (OR 2.18; 95% CI 1.21–3.94). From this model, we removed the variables which were not statistically significant predictors in the multivariate model.

## 4. Discussion

The present study revealed that over half (60.5%) of the university students consulted had reported missing at least one day of class over the previous year due to reasons related with their menstruation. This percentage was lower in women who used combined hormonal contraceptives. Dysmenorrhea, bloating, irritability, fatigue, and depression were the most commonly reported negative experiences related with menstruation. Furthermore, it was observed that the women who suffered dysmenorrhea, dizziness, nausea or vomiting, sleep disorders, and/or who felt depressed had more odds of academic absenteeism, affecting attendance to classes or clinical placements in their nursing degree studies.

The high rate of absenteeism identified during menstruation among female university students in this study is in line with previous reports conducted in other countries (although slightly higher), as analyzed in a systematic review and meta-analysis by Armour et al. [10]. Absenteeism rates were higher when associated with greater scores on the VAS scale for dysmenorrhea, these data are in agreement with former studies conducted in the Netherlands by Schoep et al., Femi Agboola et al. in Nigeria, and in Sri Lanka by Hapuarachchige et al. [24,25,26]. The high rates of absenteeism associated with dysmenorrhea and to an elevated intensity of menstrual pain could be related to an incorrect management of menstruation in Spain. As noted in former studies, most young women with dysmenorrhea do not consult a health professional, relying on self-medication [5,22]. Several authors have found that many young women with dysmenorrhea are able to endure menstrual pain and symptoms, understanding these as a typical aspect of a woman’s life, normalizing their own menstrual experiences, even if these entail limitations [27,28,29]. Ibuprofen or other over-the-counter nonsteroidal anti-inflammatory drugs are the current standard of care for dysmenorrhea, although when not taken to “stay ahead of the pain” they have less than 50% effectiveness [30,31]. Oladsu et al. highlight the possible resistance to drugs in cases of dysmenorrhea, which is estimated at approximately 18%. [32]. Hormonal contraceptives are the second choice of treatment for dysmenorrhea, and in our study, these were only taken by 11.71% of participants. Nonetheless, a reduced prevalence of dysmenorrhea and absenteeism was identified during menstruation in women who did take contraceptives, which is in line with the previous literature, however, this treatment is known to have possible adverse effects and is not accepted by all women [31,32].A number of authors consider the need to study the strategies used for treating menstrual pain and symptoms [5,22]. Although the management of menstruation is influneced by a diversity of contextual factors such as social, cultural, and economical factors, Armour et al. highlight the urgent need on a global level to provide greater information on self-care regarding the management of menstruation, as, currently, most women worldwide fail to choose the best self-care option in this matter [31]. In addition, the high rates of absenteeism identified due to menstrual pain and symptoms in our study may also be explained by the policies in place at the nursing faculty, which allow the students to make up for missed days during clinical placements, when students are unable to attend due to health issues. This situation is managed differently accross different Spanish universities, and therefore, the results may be heterogeneous. Nontheless, a rigid norm in this sense can lead to a greater presenteeism, a concept which refers to attending work or clinical placements when a person is ill or does not feel well. Presenteeism with dismenhorrea and other symptoms in nursing settings has been explored by authors such as Critz et al., who found this was related to an increased number of errors when administrating medication, a greater risk of patient falls, a decreased quality of care, and greater costs for the health institution [33]. Other authors such as Schoep et al. also studied presenteeism, noting the relevance of providing work and academic flexiblity in these matters [25].

Among the most frequent experiences or symptoms related with menstruation, we identified dysmenorrhea, bloating, irritability, and fatigue; these results are coherent with former studies [10,34,35]. Multiple previous studies already identified dysmenorrhea as being one of the most common menstrual problems among teenagers and young women, worldwide [36,37,38] and, more specifically, among Spanish university students [3]. However, bloating, irritability, and fatigue during menstruation have been less studied to date and are largely unexplored in the Spanish population, although a high prevalence of the same is found in the few studies available [10]. Nonetheless, the studies on dysmenorrhea should standardize the definition of this menstrual symptom. Thus, numerous definitions exist relating to the need for medication and the inability to function normally [39], menstrual pain during the previous six months [40], or pain which hampers activities of daily living and VAS ≥4 [41]. In addition, some authors use the term dysmenorrhea to include other experiences or symptoms which can appear during menstruation, such as tiredness, feeling depressed, nausea, or headaches [42]. However, in our study, we found that over half of women without dysmenorrhea suffered from bloating and/or irritability and, in a lesser proportion, feelings of fatigue or depression. In addition, almost 20% of these women referred absenteeism during menstruation despite not suffering from menstrual pain. In our study, besides dysmenorrhea, dizziness also increased the odds of absenteeism during menstruation. This finding is coherent with previous studies on absenteeism due to dizziness and in this context, this may be related with the fact that a large percentage of university students travel by car to the university or to their clinical placements and, therefore, dizziness can be a limitation for driving [43]. The odds of absenteeism were also higher among women who suffered from nausea and vomiting during menstruation, sleeping disorders, and/or those who felt depressed. Nausea and vomiting are considered a common reason for absenteeism in general, therefore it seems very likely that, in this context, this also contributes to the situation [44]. Regarding sleeping disorders and their repercussion on absenteeism, our results are in line with those by Woosley et al. among university students of Alabama; who, upon identifying the relationship between dysmenorrhea and absenteeism, concluded that it would be interesting to evaluate how this affects treatment for sleep disorders associated to menstruation in cases of dysmenorrhea [45]. Lastly, regarding the findings of depression during menstruation among students in our sample and its effects on absenteeism and cost-effectiveness, this has been studied in depth by Song et al. in the Chinese population, with interesting results using a mobile application for decreasing both depressive symptoms as well as dysmenorrhea in Chinese women [46].

The main strength of this study is that, for the first time, insight is provided into the situation concerning menstrual pain and symptoms related with absenteeism in Spanish university students, finding that suffering dysmenorrhea, dizziness, nausea and vomiting, sleep disorders, and/or feeling depressed during menstruation increases the odds of absenteeism. Furthermore, this study had an excellent participation rate of 96% of the eligible women in the surveyed classes.

Nonetheless, the results of the present study should be interpreted considering certain limitations related to the fact that the data were gathered using a nonvalidated questionnaire based on retrospective self-report to obtain information concerning the menstrual symptoms. On the other hand, the participants understood that the aim was to describe menstruation-related pain and other negative issues related to missing time from work or school and there is a serious risk of over-reporting as an “intent to please” the investigators. A further drawback is that, except for dysmenorrhea, we do not know how much “discomfort” or how intense the other associated symptoms were.

Other limitations of this study were that the information on absenteeism was gathered based on dichotomous yes/no questions, and no information was gathered on presenteeism nor on the specific treatment taken to relieve dysmenorrhea. Furthermore, with regards absenteeism, the study population was limited to students from a Spanish university; therefore, future multicentric studies are necessary, exploring more concrete aspects on absenteeism and presenteeism associated to menstrual pain and symptoms, academic performance, attitudes and information on menstruation, and self-care measures, both pharmacological and nonpharmacological.

## 5. Conclusions

Dysmenorrhea, dizziness, nausea and vomiting, sleep disorders, and feeling depressed in relation to menstruation affect a large percentage of healthy women, which, among nursing students in Spain, results in considerable absenteeism. Therefore, it is necessary to continue researching along these lines and to focus on the menstrual pain and symptoms which have a greatest influence on absenteeism, considering both the individual, as well as the social and economic impact.

## Figures and Tables

**Table 1:** Data sociodemographic.

Variables		*n*	%	Mean ± SD
Age				
Weight (kg)				58.69 ± 10.2
Height (cm)				164.46 ± 6.32
Body mass index (BMI)				21.67 ± 3.24
Menstrual regularity	No	200	66.9	
	Yes	99	33.1	
Menarche				12.42 ± 1.44
Days of flow				5.12 ± 1.28
Days between cycles				30.42 ± 6.29
Uses combined hormonal contraceptives	No	249	83.3	
	Yes	50	16.7	

SD—Standard deviation.

**Table 2:** Menstrual characteristics and absenteeism during menstruation.

Menstrual Characteristics		Absenteeism								*p*-Value
		No *n* (%)/ Mean ± SD				Yes *n* (%)/ Mean ± SD				
		Dysmenorrhea			*p*-Value	Dysmenorrhea			*p*-Value	
		No	Yes	Total		No	Yes	Total		
Age		20.53 ± 2.74	19.80 ± 1.72	20.16 ± 2.29	0.082	19.85 ± 1.63	20.11 ± 2.53	20.09 ± 2.47	0.715	0.799
Weight (kg)		59.98 ± 9.26	58.74 ± 12.38	59.35 ± 10.93	0.540	57.52 ± 2.53	58.31 ± 9.48	58.25 ± 9.40	0.772	0.356
Height (cm)		164.22 ± 5.31	165.02 ± 7.26	164.63 ± 6.36	0.501	165.29 ± 5.63	164.29 ± 6.38	164.36 ± 6.32	0.576	0.721
BMI		22.19 ± 2.90	21.46 ± 3.47	21.82 ± 3.21	0.213	21.06 ± 3.04	21.60 ± 3.28	21.57 ± 3.26	0.562	0.510
Menarche		12.189 ± 1.42	12.40 ± 1.45	12.49 ± 1.45	0.428	12.08 ± 1.66	12.52 ± 1.43	12.30 ± 1.43	0.285	0.253
Menstrual regularity	No	39 (50%)	39 (50%)	78 (66.1%)	0.797	11 (9%)	111 (91%)	122 (67.4%)	0.648	0.815
	Yes	19 (47.5%)	21 (52.5%)	40 (33.9%)		2 (3.4%)	57 (96.6%)	59 (32.6%)		
Days of flow	3 to 7	56 (48.7%)	59 (51.3%)	115 (97.5%)	0.539	12 (6.8%)	164 (93.2%)	176 (97.2%)	0.260	0.908
	8 or more	2 (66.7%)	1 (1.7%)	3 (2.5%)		1 (20%)	4 (80%)	5 (2.8%)		
Days between cycles	21–35 days	52 (59%)	52 (50%)	104 (88.1%)	0.616	12 (7.5%)	148 (92.5%)	160 (88.4%)	0.648	0.945
	Others	6 (42.9%)	8 (57.1%)	14 (11.9%)		1 (4.8%)	20 (95.2%)	21 (11.6%)		
Uses hormonal combined contraceptives	No	44 (48.4%)	47 (51,6%)	91 (77.1%)	0.749	11 (7%)	147 (93%)	158 (87.3%)	0.764	0.021 *
	Yes	14 (51.9%)	13 (48.1%)	27 (22.9%)		2 (8.7%)	21 (91.3%)	23 (12.7%)		

* *p* < 0.05.

**Table 3:** Absenteeism and menstrual experiences or symptoms.

Menstrual Symptoms or Experiences		Absenteeism		*p*-Value	Dysmenorrhea		Total	*p*-Value
		No	Yes		No	Yes		
Dizziness	No	113 (48.9%)	118 (51.1%)	0.000 *	70 (30.3%)	161 (69.7%)	231 (77.3%)	0.000 *
	Yes	5 (7.4%)	63 (92.6%)		1 (1.5%)	67 (98.5%)	68 (22.7%)	
Nausea and vomiting	No	109 (49.1%)	113 (50.9%)	0.000 *	67 (30.2%)	155 (69.8%)	222 (74.2%)	0.000 *
	Yes	9 (11.7%)	68 (88.3%)		4 (5.2%)	73 (94.8%)	77 (25.8%)	
Diarrhea	No	75 (44.1%)	95 (55.9%)	0.059	48 (28.2%)	122 (71.8%)	170 (56.9%)	0.036 *
	Yes	43 (33.3%)	86 (66.7%)		23 (17.8%)	106 (82.2%)	129 (43.1%)	
Sleep disorders	No	104 (46.6%)	119 (53.4%)	0.000 *	61 (27.4%)	162 (72.6%)	223 (74.6%)	0.012 *
	Yes	14 (18.4%)	62 (81.6%)		10 (13.2%)	66 (86.8%)	76 (25.4%)	
Fatigue	No	56 (63.6%)	32 (36.4%)	0.000 *	38 (43.2%)	50 (56.8%)	88 (29.4%)	0.000 *
	Yes	62 (29.4%)	149 (70.6%)		33 (15.6%)	178 (84.4%)	211 (70.6%)	
Bloating	No	23 (60.5%)	15 (39.5%)	0.004 *	19 (50%)	19 (59%)	38 (12.7%)	0.000 *
	Yes	95 (36.4%)	166 (63.6%)		52 (19.9%)	209 (80.1%)	261 (87.3%)	
Headache	No	78 (47.6%)	86 (52.4%)	0.002 *	56 (34.1%)	108 (65.9%)	164 (54.8%)	0.000 *
	Yes	40 (29.6%)	95 (70.4%)		15 (11.1%)	120 (88.9%)	135 (45.2%)	
Irritability	No	43 (60.6%)	28 (39.4%)	0.000 *	28 (39.4%)	43 (60.6%)	71 (23.7%)	0.000 *
	Yes	75 (32.9%)	153 (67.1%)		43 (18.9%)	185 (81.1%)	228 (76.3%)	
Depression	No	63 (52.9%)	56 (47.1%)	0.000 *	41 (34.5%)	78 (65.5%)	119 (39.8%)	0.000 *
	Yes	55 (30.6%)	125 (69.4%)		30 (16.7%)	150 (83.3%)	180 (60.2%)	
Dysmenorrhea	No	58 (81.7%)	13 (18.3%)	0.000 *			71 (23.7%)	
	Yes	60 (26.3%)	168 (73.7%)				228 (76.3%)	

* *p* < 0.05.

**Table 4:** Regression on absenteeism during menstruation and menstrual experiences or symptoms.

Menstrual Experiences or Symptoms	β	SE	df	*p*-Value	OR	95% CI
Dizziness	1.573	0.515	1	0.002 *	4.82	1.76–13.23
Nausea and vomiting	1.255	0.430	1	0.004 *	3.51	1.51–8.15
Sleep disorders	1.081	0.384	1	0.005 *	2.95	1.39–6.25
Depressed	0.779	0.302	1	0.010 *	2.18	1.21–3.94
Dysmenorrhea	1.938	0.367	1	0.000 *	6.95	3.39–14.25
Constant	−2.213	0.381	1	0.000 *	0.107	

* *p* < 0.05. Nagelkerke R^2^ = 0.44. β—Beta; SE—Standard error; df—Degrees of freedom; OR—Odds ratio; CI—Confidence interval.

## Supplementary Materials

The following are available online at https://www.mdpi.com/1660-4601/17/1/53/s1, Supplementary file 1: English translation of the study questionnaire.
