# Supplementary material for: Absenteeism during Menstruation among Nursing Students in Spain
Source: Int J Environ Res Public Health. 2019 Dec 19;17(1):53. doi: 10.3390/ijerph17010053 (PMC6981649; doi:10.3390/ijerph17010053)
Supplement: Supplementary file 1 [file ijerph-17-00053-s001.pdf]

Supplementary file 1. English translation of the study questionnaire.

1. Age:.....years
2. Weight:...... kg
3. Height:...... cm
4. Menarche:.....years
5. Is your menstruation regular? YES NO
6. How many days do you usually spot during menstruation? …. days
7. How many days usually pass between the first day of your period and the first day of the next?.....days
8. What contraceptive method do you currently use?

NONE / CONDOM/COMBINATION PILL/ MINIPILL/IMPLANT/RING/PATCH/ COPPER DIU/ HORMONAL DIU/ DEFINITIVE METHOD

9. Do you have any diagnosed gynaecological disease: endometriosis, polycystic ovaries...? YES/ NO

10. Do you have a diagnosed chronic non-gynecological disease? YES/ NO

11. Do you have menstrual pain at least 3 cycles a year? YES / NO

If the answer is yes, indicate the intensity of the pain on a scale from 0 to 10 in which 0 is no pain and 10 is the maximum possible pain.

12.-In most of your periods:

- Do you have nausea and/or vomiting during menstruation? YES NO
- Do you have diarrhea during menstruation? YES NO
- Do you have swelling during menstruation? YES NO
- Do you feel irritable during menstruation? YES NO
- Do you feel depressed during menstruation? YES NO
- Do you have a headache during menstruation? YES NO
- Do you experience dizziness during menstruation? YES NO
- Do you experience fatigue during menstruation? YES NO
- Is your sleep disturbed during menstruation? YES NO

13. Has menstrual pain and/or symptoms occurring during menstruation caused you to miss school or clinical practice at any time in the past year? YES /NO
